# Impact of community-based presumptive chloroquine treatment of fever cases on malaria morbidity and mortality in a tribal area in Orissa State, India

**DOI:** 10.1186/1475-2875-7-75

**Published:** 2008-05-05

**Authors:** Lalit K Das, Purushothaman Jambulingam, Candasamy Sadanandane

**Affiliations:** 1 Vector Control Research Centre, Indian Council of Medical Research, Indira Nagar, Pondicherry-605006, India

## Abstract

**Background:**

In the Global Strategy for Malaria Control, one of the basic elements is early detection and prompt treatment of malaria cases, especially in areas where health care facilities are inadequate. Establishing or reviving the existing drug distribution centers (DDC) at the peripheral levels of health care can achieve this. The DDCs should be operationally feasible, acceptable by community and technical efficient, particularly in remote hard-core malaria endemic areas.

**Methods:**

Volunteers from villages were selected for distribution of chloroquine and the selection was made either by villagers or head of the village. The services of the volunteers were absolutely free and voluntary in nature. Chloroquine was provided free of charge to all fever cases. The impact was evaluated based on the changes observed in fever days, fever incidence, parasite incidence and parasite prevalence (proportion of persons harbouring malaria parasite) in the community. Comparisons were made between 1st, 2nd and 3rd year of operation in the experimental villages and between the experimental and check areas.

**Results:**

A total of 411 village volunteers in 378 villages in the experimental community health center with a population of 125,439 treated 88,575 fever cases with a mean annual incidence of 331.8 cases per 1,000 population during the three-year study period. The average morbid days due to fever (AFD) was reduced to 1.6 ± 0.1 from 5.9 ± 2.1 in the experimental villages while it remained at 5.0 ± 1.0 in the check villages. There was a significant reduction, (p < 0.05) in Annual Fever Incidence (AFI) in the experimental hilltop and foothill villages in comparison to check villages. The change in Annual Parasite Incidence (API) was, however, not statistically significant (p > 0.05). In plain villages that were low endemic, the reductions in AFI and API in experimental villages were statistically significant (p < 0.05). There was significant reduction in the parasite prevalence in high endemic villages of the experimental area both during 2^nd ^and 3^rd ^year when compared with the check area (p < 0.05) but no such reduction was observed in low endemic areas (p > 0.0.5). Mortality due to malaria declined by 75% in the experimental villages in the adult age group whereas there was an increasing trend in check villages.

**Conclusion:**

The study demonstrated that a passive chloroquine distribution system operated by village volunteers in tribal areas is feasible and effective in reducing malaria-related morbidity and mortality.

## Background

The present strategies for malaria control vary depending up on the epidemiological situations. Until a safe, effective and affordable vaccine against malaria is available, its control will depend on antimalarial drugs and measures for reduction in man-vector contact. Malaria remains a major public health problem in India and about three million clinical cases are recorded every year. The incidence of *Plasmodium falciparum *has shown an increasing trend, a major proportion of cases being recorded from hilly and forested regions of the country [1]. Health care facilities are inadequate in these areas and death from malaria is common [2]. The Government of India has implemented a three-pronged strategy, under the Modified Plan of Operation (MPO) in 1977, with an emphasis on early case detection and treatment to reduce mortality from malaria. Drug Distribution Centers (DDCs) were established at village level and they are manned by a village health guide (VHG) [1]. The programme was integrated with the primary health care delivery system. The functioning of DDCs deteriorated due to lack of proper supervision, short supply of drugs, influence of local politics in selecting the VHGs, and the poor and irregular remuneration of VHGs (Rs.50, approx. US $1, per month). This resulted in defunct DDCs. Neither the functioning of DDCs and VHGs were evaluated on time nor any modifications to operational strategies were made for effective implementation.

In the Global Strategy for Malaria Control, one of the basic elements is early detection and prompt treatment [3], especially in areas where health care facilities are inadequate. National Programme in India is now laying emphasis on reviving the drug distribution system under various health schemes. Therefore, there was a need to establish DDCs or revive the existing ones, evaluate the operational feasibility, community acceptability and technical efficiency of the system, particularly in remote areas. This communication presents the results of a study undertaken to assess the feasibility of establishing drug distribution centres through village volunteers in a tribal area, where health-seeking practice of the community has been poor. The impact of treatment of fever cases with chloroquine (10 mg/kg. body wt., single dose) on morbidity, mortality and parasite prevalence in community were assessed.

## Materials and methods

### Study villages

**Figure 1:**
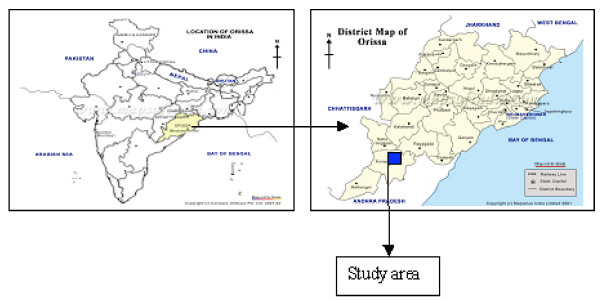
Map of the study area.

The strategy was tested in 378 villages under Borigumma Community Health Centre (CHC) in southern part of Koraput district (17° 50' and 20°30'N and 81° 27' and 84°10'E), Orissa State, India (Figure 1). The CHC is the largest in the state of Orissa catering to the basic health care needs of 125,439 population. The topography and dynamics of malaria transmission in the area have already been described [4]. Villages situated on hilltops and at the foot of hills are hyper-endemic for malaria and experience perennial transmission. Villages situated on plain lands and riverbanks are hypo-endemic for malaria and experience seasonal transmission in rainy season [4,5]. The houses have thatched roofing and mud plastered walls and are usually in-groups to form a village. Majority of the population is of tribal aborigines, who are economically poor. The men mainly cultivate paddy and women collect forest products, usually sal leaves and firewood. The health care facilities are poor and the first " medical" assistance is from traditional healers [6]. Under the National Anti-Malaria Programme, apart from routine surveillance and treatment, residual insecticide spraying was done in areas where annual parasite incidence was >10. Villages adjacent to high endemic villages, under Dasmantpur CHC with a population of about 66,500 and villages adjacent to low endemic villages under Kotpad CHC with a population of about 76,400 were selected as corresponding check areas for comparison.

### Choroquine sensitivity status of *Plasmodium falciparum *in the study area

The major malaria species of the study area is *P. falciparum *(85–90%)[5]. The parasite is sensitive to chloroquine in the study area as evidenced by both in vivo and in vitro methods [6,7].

### Selection of village volunteers

One volunteer from each village was selected for distribution of chloroquine and the selection was made either by villagers or head of the village. The volunteers were imparted training on diagnosis based on the symptoms of malaria and on chloroquine administration as prescribed in the National Anti-Malaria Programme through group discussion sessions and demonstrations.

Volunteers were advised to provide chloroquine to those patients who approach them for treatment and to fill up a 'fever treatment sheet' indicating the name, age, sex, number of days suffering from fever and the number of chloroquine tablets given. Those volunteers with no educational background were supplied with pre-packed chloroquine tablets in colored disposable plastic pouches for different age classes. The number of suspected malaria cases treated by age class was counted from number and type of pouches available with the volunteer at any point of time. Chloroquine was provided free of cost to patients. The volunteers were neither paid nor remunerated in kind.

The fever treatment sheets were collected from the volunteers after replenishment of chloroquine tablets through nine field workers located at different centres of the CHC area at fortnightly interval. Some Anganwadi workers of Integrated Child Development Scheme (ICDS) of the state Government also served as chloroquine distributors in addition to village volunteers to have better accessibility of all sections of people of the village.

### Evaluation

The impact was evaluated based on the changes observed in fever days (the average number of days a patient suffered from fever before treatment), fever incidence (number of fever cases/year/1,000), malaria parasite incidence (number of microscopically proved malaria cases/year/1,000) and parasite prevalence (proportion of persons harbouring malaria parasite) in the community. Fever incidence in experimental area was calculated from the total number of cases receiving treatment from DDCs or Anganwadi centres. Malaria incidence was determined from the fortnightly fever surveillance carried out from 47 randomly selected villages (10% of the total population) in the experimental area. The finger prick blood slides were stained with Giemsa and 100 fields in each thick film were examined with 100× magnification [8]. Surveillance records of the CHC were used to determine fever and malaria incidence in the check area. Cross sectional sample blood surveys were carried out following identical methodologies in both the experimental and check areas at pre-intervention and six monthly during intervention (cold and dry season) to know the impact on the malaria parasite prevalence in the community. The villages to be sampled were selected following random number sampling design so as to cover 10 % of the total population from each endemic zone.

The number of fever cases not taking treatment from volunteers was identified through door-to-door surveys in randomly selected villages every fortnight. The patients were interviewed to know the reason for not seeking treatment from DDCs.

Comparison of impact of fever incidence, parasite incidence and parasite prevalence was made between 1st, 2nd and 3rd year of operation in the experimental villages and between the experimental and check CHCs. The odds ratio test was used to compare the change in fever and parasite incidence between experimental and check villages during 1^st ^and after 3^rd ^year of intervention.

Information on the deaths attributed to malaria was collected from the records available at the check and experimental CHCs. However, there is a wide spectrum severe and complicated manifestation of falciparum malaria reported from this area [9].

## Results

### Performance of volunteers

Table 1 shows the number of village volunteers and Anganwadi workers as chloroquine distributors in the two endemic areas of the study CHC. A total of 411 volunteers (281 village volunteers and 130 Anganwadi Workers) participated in chloroquine distribution in the 378 villages. Out of 281 village volunteers, 88.3% was males. The majority of volunteers (68%) was either small farmers or agricultural labourers earning less than Rupees 1,000/- (approximately 20 US$) per month. The remaining included petty shop owners, students, government employees, contractors, community leaders, former village health guides, Ayurvedic practitioners and a traditional healer. A total of 24 (8%) had no formal education. Among the literates, 35% had elementary education and the remaining had education up to 10^th ^standard or more. Most of the volunteers were family heads (75%).

**Table 1:** Population, volunteers, DDCs and FTDs in high and low endemic villages

Endemicity	No. Villages	Popn.	Anganwadi workers & centres	No. of * Village Volunteers	Total no. of DDCs	No. of FTDs
High	112	27332	26	88 (24)	114	16
Low	266	98107	104	193	297	31
**Total**	**378**	**125439**	**130**	**281 (24)**	**411**	**47**

*The discrepancy in the numbers of villages and DDCs is because of some villages having more than one DDC, manned by Anganwadi worker and village volunteer.

DDCs- Drug Distribution Centres.

FTDs- Fever Treatment Depots.

Figure in the parentheses indicates number of illiterate volunteers.

Majority of the volunteers administered chloroquine as per the age class dosage schedule and maintained records properly. About 10% of the volunteers were replaced at different points of time as they were losing interest and were not performing well. The majority (64%) of volunteers reported having participated in drug distribution for social recognition.

The volunteers treated 88,575 fever cases during the 3-year study period, 39,301 (mean 492 ± 76 cases per annum per 1,000 population) from high endemic and 49,274 (mean 236 ± 142 cases per annum per 1,000 population) from low endemic experimental villages. In check areas, 26,657 (mean 137 ± 12) fever cases were treated from high endemic villages and 28,354 (mean 120 ± 29) from low endemic villages. About 14.5% of fever cases did not receive treatment from DDCs (Table-2) of which 17.9% did so due to mere negligence, whereas some 13.8% had no faith in the DDCs, probably because local persons manned them.

**Table 2:** Reported reasons for not receiving treatment from DDCs

No. of villages/DDCs surveyed	457
No. of fever cases interviewed	3233
No. of cases not received treatment from DDCs	468(14.5%)
**Reasons**	
1. Unaware of DDCs	61(13.0)
2. No relief with chloroquine treatment	38 (8.1)
3. Prefer injections	35 (7.5)
4. Unwilling to get treatment for infants	42 (8.9)
5. Negligence, waiting to be relieved from fever	84 (17.9)
6. Prefer to take medicines from PHC/ANM/MPW	15 (3.2)
7. Absence of volunteers at time of visit	46 (9.8)
8. Unwilling to take treatment for women who have delivered	35 (7.5)
9. Belief that tablets reduce fever in day time only	15 (3.2)
10. Volunteer was from a lower caste	8 (1.7)
11. No one to help to approach DDCs	4 (0.8)
12. No trust in DDCs	65 (13.8)
13. Prefer sugar coated tablets	8 (1.7)
14. No stock of tablets with the volunteer at the time of visit	4 (0.8)
15. Unspecified	7 (1.5)

PHC – Primary Health Centre.

ANM – Auxiliary Nurse Mid-wife.

MPW – Multi Purpose Worker

### Impact on fever incidence

**Figure 2:**
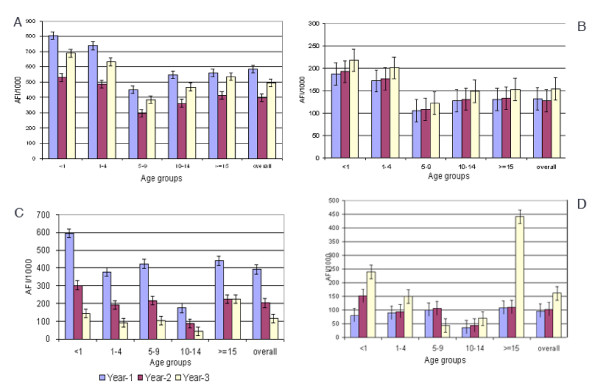
Age group wise Annual Fever Incidence (A-high endemic, experimental villages, B-high endemic check villages, C- low endemic, experimental villages, D- low endemic check villages).

The age wise and overall annual fever incidence (AFI) in high endemic villages of the experimental area (Figure 2A) and check areas (Figure 2B) shows that the AFI was reduced by 31.7% in the 2nd year with a subsequent increase by 24.1% during the 3rd year in the experimental villages, whereas the reduction was 3.05% in the 2nd year with an increase of 20.5% during the 3rd year in the check area. The overall reduction observed in AFI at the end of 3rd year in the experimental villages was significantly higher (χ^2 ^= 520.03, P < 0.05), when compared to the check villages. The reduction in AFI during the 2nd year and subsequent increase during the 3rd year was observed in all age classes and in all months in the experimental area.

**Figure 3:**
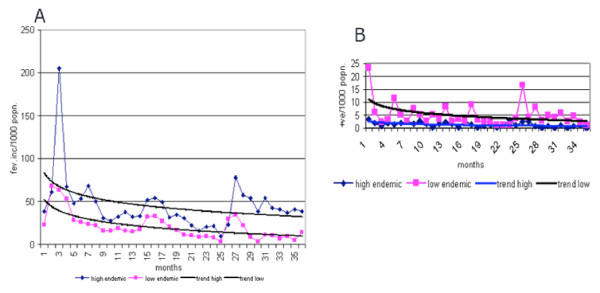
Trends in month-wise fever and parasite incidence in the high and low-endemic villages of the experimental area (A-Fever incidence, B-Parasite incidence).

The age wise and overall annual fever incidence (AFI) in low endemic villages of the experimental area (Figure 2C) and check areas (Figure 2D) shows that in low endemic villages of the experimental area, the AFI was reduced by 48.4% during the 2nd year with a further reduction by 43.2% during the 3rd year in the experimental area, whereas there was an increase by 6.25% and 58.6% during the corresponding years in the check area. The overall reduction observed in AFI at the end of 3rd year in the experimental villages was significantly higher (χ^2 ^= 5712.75, P < 0.05) when compared to the check villages. The AFI declined in all age classes during the 2nd year of operation with further reduction during the 3rd year, except in the adult age class and in all months in the experimental area (Figure 3A).

### Impact on parasite incidence

In high endemic villages, the API reduced by 63.1% during the 2nd year followed by an increase of 66.6% during the 3rd year in experimental villages, whereas the reduction was 21.2% followed by an increase of 24% during the corresponding period in the check villages. The overall reduction observed in API at the end of 3^rd ^year was not significantly different (χ^2 ^= 1.46, P > 0.05)) between the experimental and check villages. No definite month-wise pattern was observed in monthly parasite incidence in the high endemic villages of the experimental area (Figure 3B).

In low endemic villages, the API was reduced by 56.8% during the 2nd year in experimental villages, whereas in the control villages the percentage of reduction was only 3.4%. However, during the 3rd year there was a marginal increase of 14% in API in the experimental villages when compared to an increase of 79.9% in the control villages. The overall reduction observed in API at the end of 3^rd ^year was significantly different (χ^2 ^= 40.14, P < 0.05)) between the experimental and check villages. Though no definite month-wise pattern was observed in monthly parasite incidence, there was declining trend in the low endemic villages of the experimental area (Figure 3B).

### Impact on parasite prevalence

**Figure 4:**
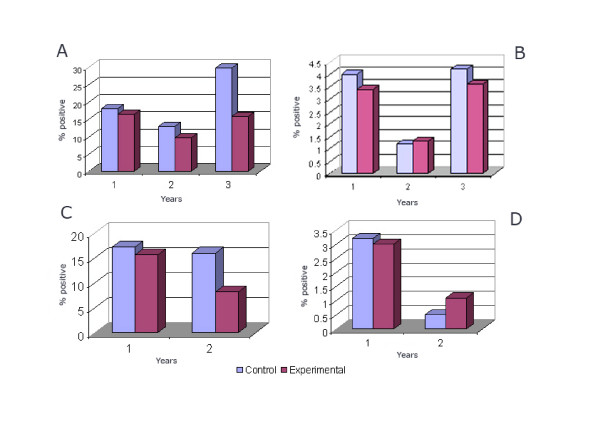
Parasite prevalence in the study area (A-high endemic, cold season, B-low endemic, cold season, C- high endemic, dry season, D- low endemic, dry season).

The pre-intervention parasite prevalence was 17.2% and 15.6% in high endemic check and experimental areas respectively. The survey was carried out in the month of May (dry season). There was marginal increase in the parasite prevalence during the cold season (month of December) during the first year in both the check (18%) and experimental (20.7%) areas. However, there was significant reduction (P < 0.05) in the prevalence in the experimental area both during dry and cold seasons of 2nd and 3rd year when compared with the check area (Figures 4A and 4C).

The pre-intervention parasite prevalence in dry season (month of May) was 3.2% and 3% in low endemic check and experimental areas respectively. There was a marginal increase in the parasite prevalence during the cold season (month of December) during the 1^st ^year in both the check (4%) and experimental (3.4%) areas. However, there was no significant reduction (P > 0.05) in the prevalence in the experimental area both dry and cold during 2^nd ^and 3^rd ^year when compared with the check area (Figures 4B and 4D).

### Impact on morbidity days

The average number of fever days (AFD) during the pre-control period was 5.9 and 4.8 in the experimental and control villages respectively. It reduced to 1.6 in the experimental study area where as it remained at five in the check areas.

### Impact on mortality due to malaria

Data on deaths confirmed to be due to malaria during the study period in check and experimental CHCs are given in Table 3. During the year preceding the study, a total of nine deaths were recorded in the check area and four in the experimental area. During the three years of intervention, a total 53 deaths occurred in the check villages whereas, in contrast to three deaths in the experimental villages. The reduction could be observed in the 10–14 and >= 15 year age class. No conclusion could be drawn on the impact on younger age classes since no deaths was recorded in the experimental CHC in these age classes during the pre-intervention year.

**Table 3:** Age group-wise number of deaths due to malaria in the high endemic experimental and check CHCs.

Age group (In years)	Pre-intervention period (one year)		Intervention period Cumulative (for 3 years)		Post-intervention period (one year)	
	Check	Experimental	Check	Experimental	Check	Experimental

<1	1	0	9	0	2	0
1–9	4	0	27	0	14	0
10 & above	4	4	17	3	11	1
Total	9 (1.4)	4 (1.5)	53 (8.2)	3 (1.1)	27 (4.2)	1 (0.4)

Figures in the parentheses indicate the number per 10,000 population.

## Discussion

This was the first study involving village volunteers for the treatment of fever cases in India covering a population of more than one lakh (one hundred thousand) in a tribal area known to be hyper-endemic for *P*. *falciparum *malaria for decades. The study has shown that trained community volunteers were able to provide treatment to suspected malaria cases. It has also demystified the misconception that, in tribal areas, people have faith in traditional treatments only. Many of them adopt their native remedies and witchcraft, as the chloroquine treatment was not readily available to them. The timely treatment provided by community volunteers was acceptable to the community. Therefore, it can be concluded that it is feasible to establish and sustain DDCs in tribal areas, provided proper training and logistic support is ensured. Malaria surveillance and treatment using community volunteers had been successful in different epidemiological settings [10-13]. However, in the Philippines, the involvement of volunteers could not contribute optimally because of inadequate training, lack of logistic support, poor sustainability of motivational schemes and lack of community support.

Data on malarial mortality suggest that there was a reduction of malarial deaths in the experimental villages in comparison to the check villages. The impact was visible in the 10–14 and >= 15 year age classes. Although the results on malaria deaths have to be judged with caution because of under-reporting, they give, nevertheless, a definite trend of lower death rates attributable to fever in the study area in comparison to control area. Data from hospital admissions of severe cases indicated that the proportion of patients admitted with history of fever reduced from 76% (one out of 13) at the beginning of the study to 33% within two years (one out of 30) and thus suggesting that many cases were treated at the village. The present study also showed that in high endemic villages the strategy could not reduce malaria incidence and prevalence. The reduction during the 2^nd ^year was most probably due to the additional effect of residual insecticide spraying with DDT by the state government. Additionally, vector control measures are required in these areas for transmission reduction.

In low endemic villages, treatment of suspected fever cases through DDCs reduced malaria incidence, but not prevalence during three-year study period. It may take a longer period to liquidate the parasite reservoir in these areas. However, since only 19% of the total fever cases recorded in the villages were malaria positives, opening DDCs in these low endemic areas may result in excessive distribution of chloroquine. Therefore, emphasis could be on opening fever treatment centres apart from the regular fever surveillance and prompt radical treatment of parasite positive cases. Presumptive treatment of fever cases with chloroquine of 10 mg/kg single dose has a definite effect in reducing the morbid days due to fever and reduction in the number of malaria related deaths and villagers were willing to participate in the treatment system provided the supply of chloroquine was on time and regular.

## Competing interests

The authors declare that they have no competing interests.

## Authors' contributions

**LKD**: Project formulation, implementation, data analysis, manuscript preparation.

**PJ**: Project formulation, study design, review, manuscript preparation.

**CS**: Project implementation.

All authors read and approved the final manuscript.

## References

[B1] Sharma RS, Sharma GK, Dhillon GPS (1996). Epidemiology and control of malaria in India.

[B2] Rajagopalan PK, Das PK (1988). Primary health care. Theory and practice in the Indian context. Indian Council of Medical Research Bulletin.

[B3] World Health Organization (1993). Implementation of the Global Malaria Control Strategy Report of a WHO study group on the implementation of the global plan of action for malaria control WHO Technical Report Series.

[B4] Rajagopalan PK, Das PK, Jambulingam P, Mohapatra SS, Gunasekaran K, Das LK (1990). Parasitological aspects of malaria persistence in Koraput district of Orissa, India. Indian J Med Res.

[B5] Jambulingam P, Mohapatra SS, Goverdhini P, Das Lalit Kumar, Manoharan A, Pani SP, Das PK (1990). Microlevel epidemiological variations in malaria & its implications on control strategy. Indian J Med Res.

[B6] Mohapatra SS, Das LK, Pani SP (1989). Chloroquine sensitivity of *P. falciparum *in Koraput district, Orissa. Indian J Malariol.

[B7] Das LK, Sahu SS (1996). A short note on present trend of chloroquine sensitivity of *P. falciparum *in Malkangiri district of Orissa state. Indian J Malariol.

[B8] Bruce Chwatt LJ (1985). Essential Malariology.

[B9] Das LK (2000). Spectrum of severe and complicated manifestations of *falciparum *malaria in Koraput district, Orissa state. The National Medical Journal of India.

[B10] Ghebreyesus TA, Alemayehu T, Bosman A, Witten KH, Teklehaimanot A (1996). Community participation in malaria control in Tigray region, Ethiopia. Acta Trop.

[B11] Rahman SH, Mohamedani AA, Mirgani EM, Ibrahim AM (1996). Gender aspects and women's participation in the control and management of malaria in Sudan. Soc Sci Med.

[B12] Ruebush II, Trenton K, Zeissig, Rodolfo, Robert KleinE, Hector GodoyA (1992). Community participation in malaria, surveillance and treatment II. Evaluation of volunteer collaborator network of Guatemala. Am J Trop Med Hyg.

[B13] Spencer HC, Kaseje DC, Roberts JM, Huong AY (1987). Consumption of chloroquine phosphate provided for treatment of malaria by volunteer village health workers in Saradidi, Kenya. Ann Trop Med Parasitol.

